# Supplementary material for: Frontal polymerization-triggered simultaneous ring-opening metathesis polymerization and cross metathesis affords anisotropic macroporous dicyclopentadiene cellulose nanocrystal foam
Source: Commun Chem. 2022 Oct 7;5:119. doi: 10.1038/s42004-022-00740-1 (PMC9814902; doi:10.1038/s42004-022-00740-1)
Supplement: Supplementary file 2 — Supplementary Information File [file 42004_2022_740_MOESM2_ESM.pdf]

Supplementary Information

Frontal Polymerization-triggered Simultaneous Ring-opening Metathesis Polymerization and Cross Metathesis Affords Anisotropic Macroporous Dicyclopentadiene Cellulose Nanocrystal Foam

Jinsu Park[a], *Seung-Yeop Kwak*[a,b,c*]

a Department of Materials Science and Engineering, Seoul National University, 1 Gwanak-ro, Gwanak-gu, Seoul, 08826, South Korea

b Research Institute of Advanced Materials (RIAM), Seoul National University, 1 Gwanak-ro, Gwanak-gu, Seoul, 08826, South Korea

c Institute of Engineering Research, Seoul National University, 1 Gwanak-ro, Gwanak-gu, Seoul, 08826, South Korea

* Corresponding Author: **Seung-Yeop Kwak**

Tel: +82-2-880-8365 (2257); Fax: +82-2-885-1748; Email: sykwak@snu.ac.kr

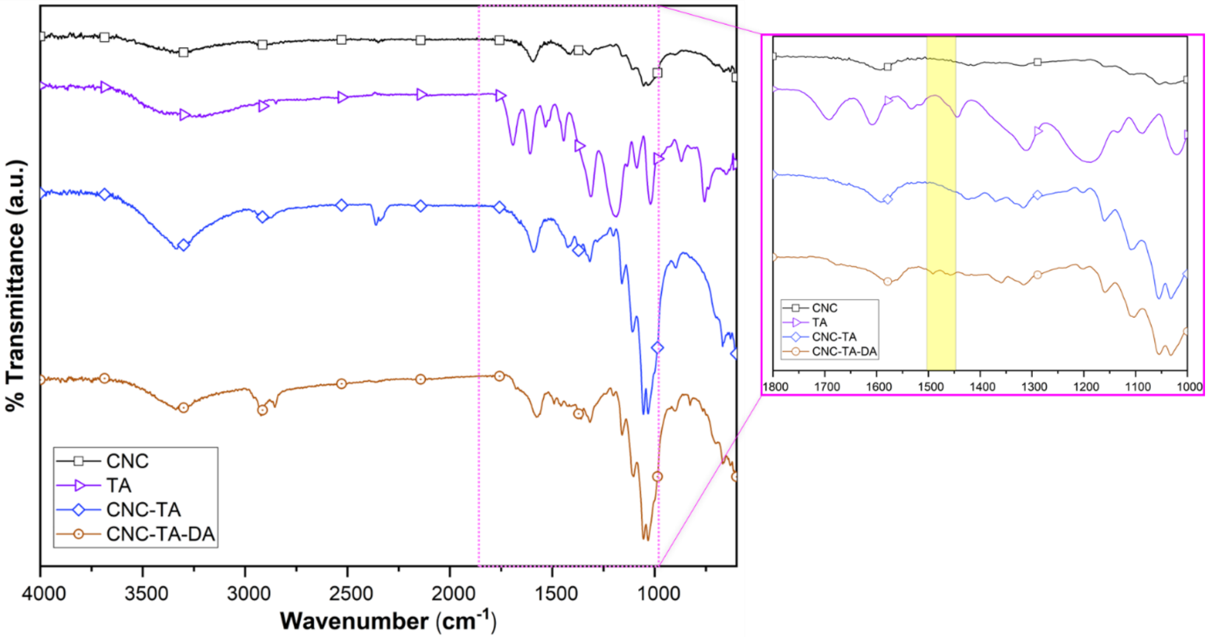

Supplementary Figure 1. Attenuated total reflectance-fourier-transform infrared spectroscopy (ATR-FTIR) spectra of unmodified CNC, tannic acid (TA), and CNC-TA-Decylamine showed a complete coating of CNC with decylamine moiety for the initial FP experiment. Secondary -NH group bending was observed at approximately 1490 cm^{-1}.[37]

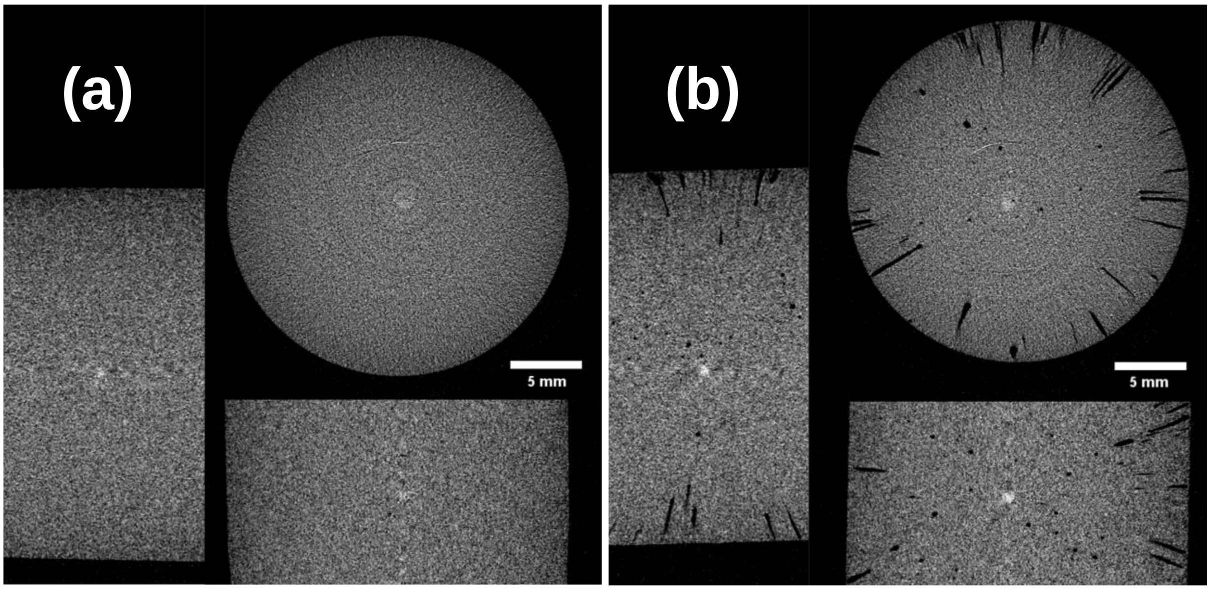

Supplementary Figure 2. μ-CT images of **(a)** neat DCPD monolith and **(b)** DCPD/1wt% CTD monolith. Both samples were frontally cured in a 20 mL vial.

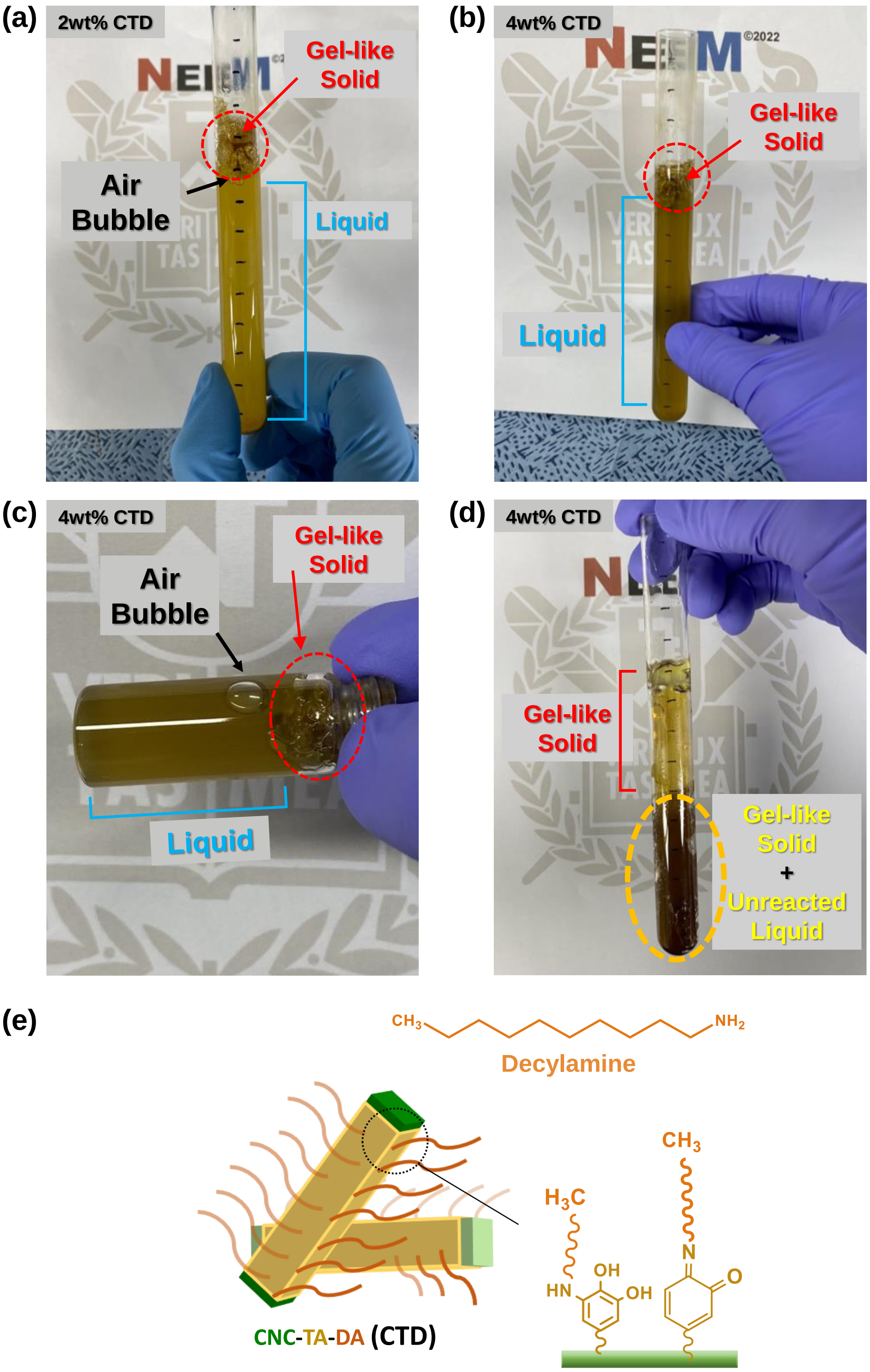

Supplementary Figure 3. FP of DCPD/CTD were performed at **(a)** 2wt% CTD and **(b – d)** 4wt% CTD. The same thermal stimulus used in the FP reactions of neat DCPD, DCPD/AC, and DCPD/0.5 – 1wt% CTD could not initiate the reaction of 2 – 4wt% CTD through heating of the bottom of the glass. When put the soldering iron in direct contact with the liquid monomer/CTD mixture, gel-like solid was formed; however, the reaction did not propagate thus leaving the unreacted portion in liquid form. **(e)** A schematic of the generated CTD material may suggest that that higher wt% of the non-allylic moiety of CTD hindered an uninterrupted FP reaction to be initiated.

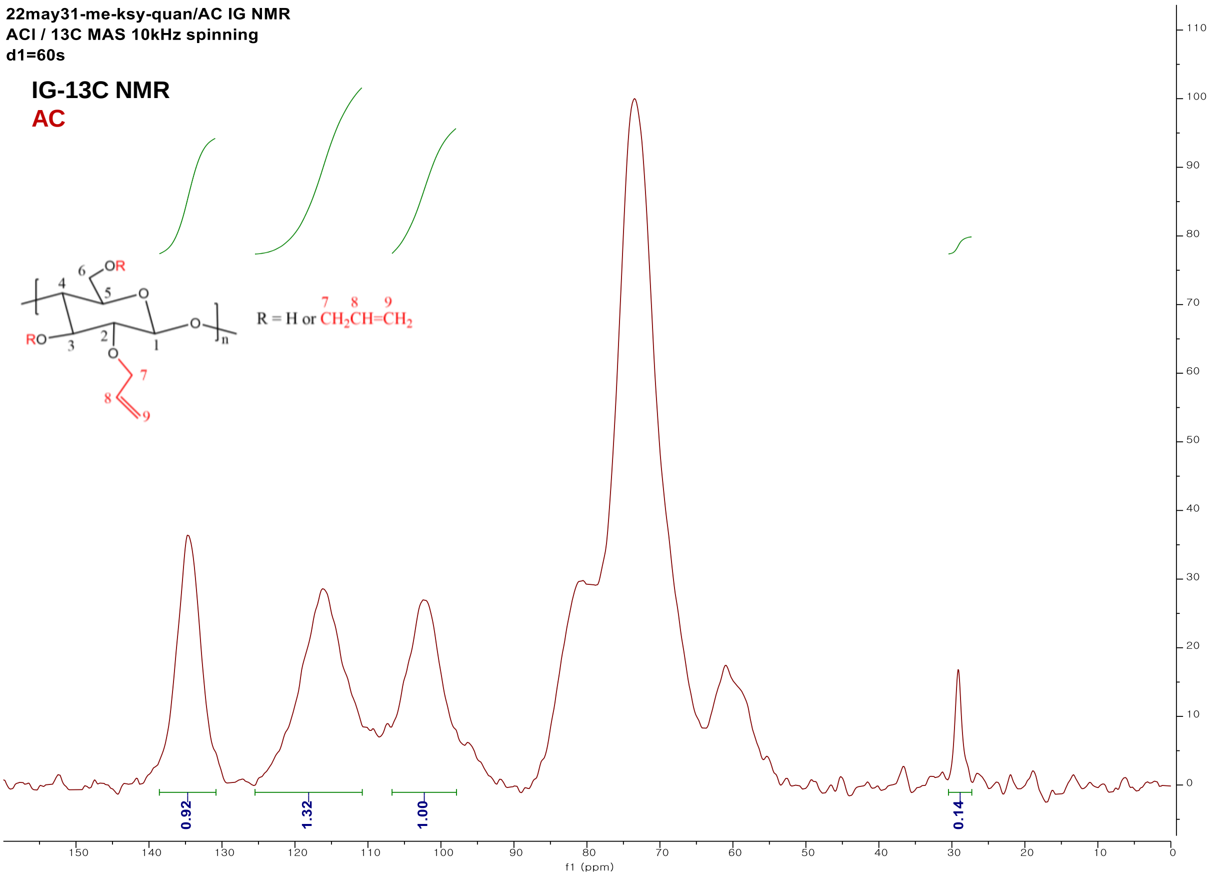

Supplementary Figure 4. Solid state ^{13}C Inverse -gated NMR spectrum of AC. Peak integration values of the carbon peaks pertaining to the allylic group assigned to C7, C8, and C9 were 0.14, 0.92, and 1.3, respectively.

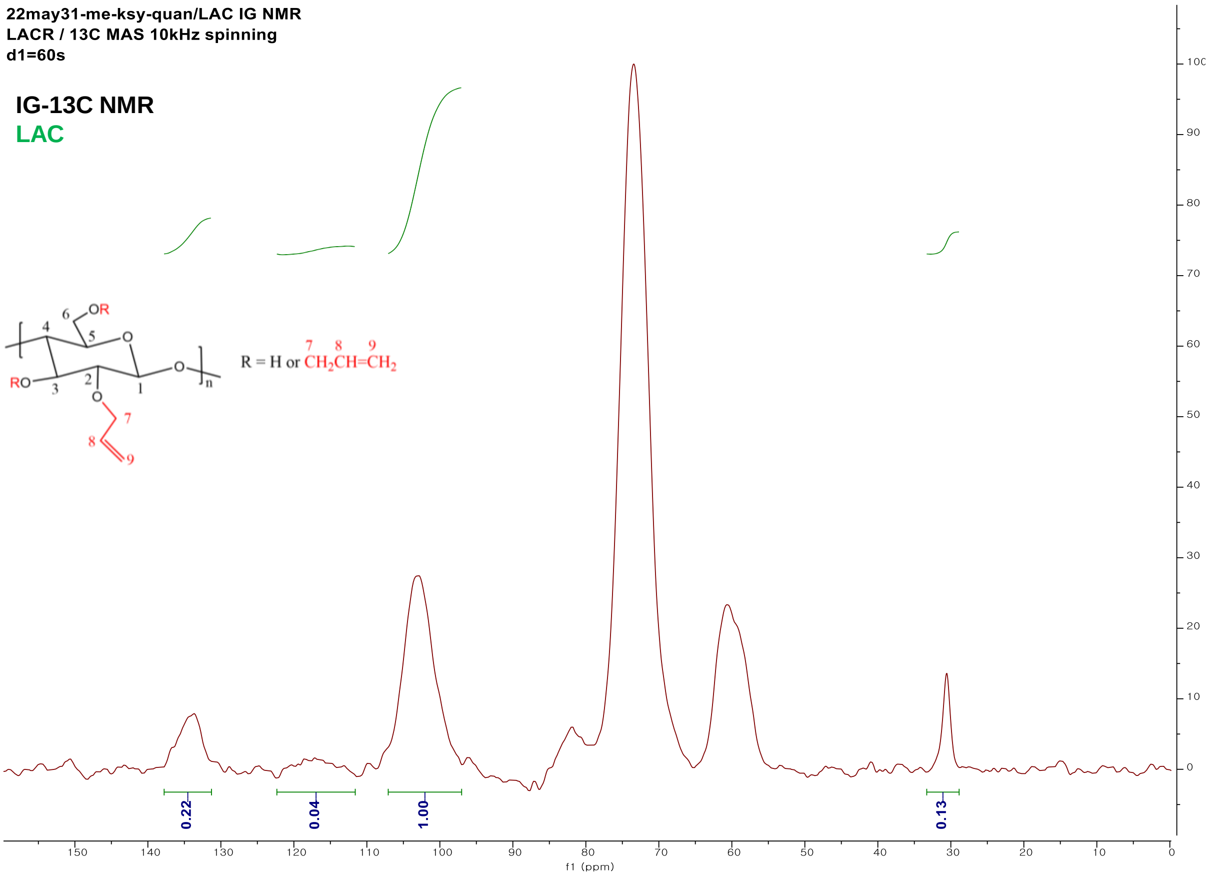

Supplementary Figure 5. Solid state ^{13}C Inverse -gated NMR spectrum spectrum of LAC. Peak integration values of the carbon peaks pertaining to the allylic group assigned to C7, C8, and C9 were 0.13, 0.22, and 0.044, respectively.

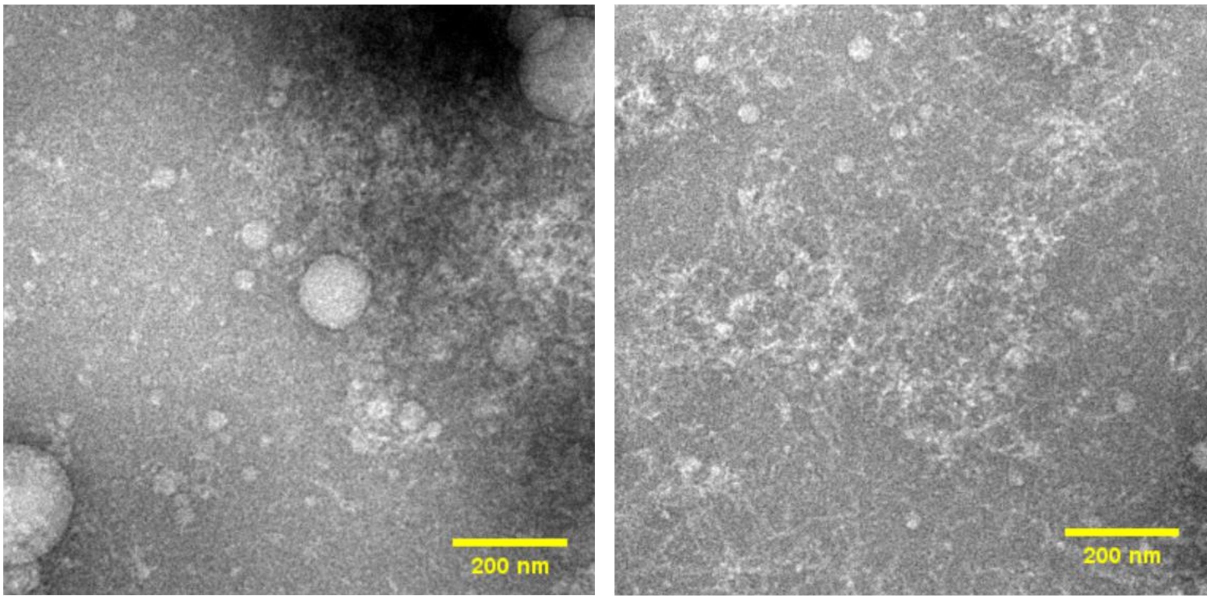

Supplementary Figure 6. TEM images showing dispersion of AC conducted using (left) ultrasonic bath only and (right) both a probe ultrasonicator (amp. = 25% for 20 min, amp. = 35% for 10 min) and an ultrasonic bath (10 min) obtained at 200 nm.

Supplementary Table 1. Dimensions of the DCPD/AC foams produced via FP.

AC	0 wt%	0.5 wt%	1 wt%	2 wt%	4 wt%
**Diameter (cm)**	1.293	1.289	1.294	1.290	1.297
**Length (cm)**	9.917	11.658	14.949	14.848	14.94
**Weight (m) (g)**	12.95	12.72	11.81	9.46	8.15
**Density (ρ) (cm^3 g^{-1})**	0.9942	0.8358	0.6005	0.4876	0.4127
**Volume Fraction (ɸ)**	1.000	0.8407	0.6040	0.4904	0.4151

Supplementary Table 2. Dimensions of the DCPD/LAC foams produced via FP.

LAC	0 wt%	0.5 wt%	1 wt%	2 wt%	4 wt%
**Diameter (cm)**	1.293	1.291	1.292	1.287	1.283
**Length (cm)**	9.917	10.287	10.603	12.063	13.116
**Weight (m) (g)**	12.95	12.88	12.83	12.55	12.51
**Density (ρ) (cm^3 g^{-1})**	0.9942	0.9567	0.9231	0.8000	0.7378
**Volume Fraction (ϕ)**	1.000	0.9623	0.9285	0.8047	0.7421

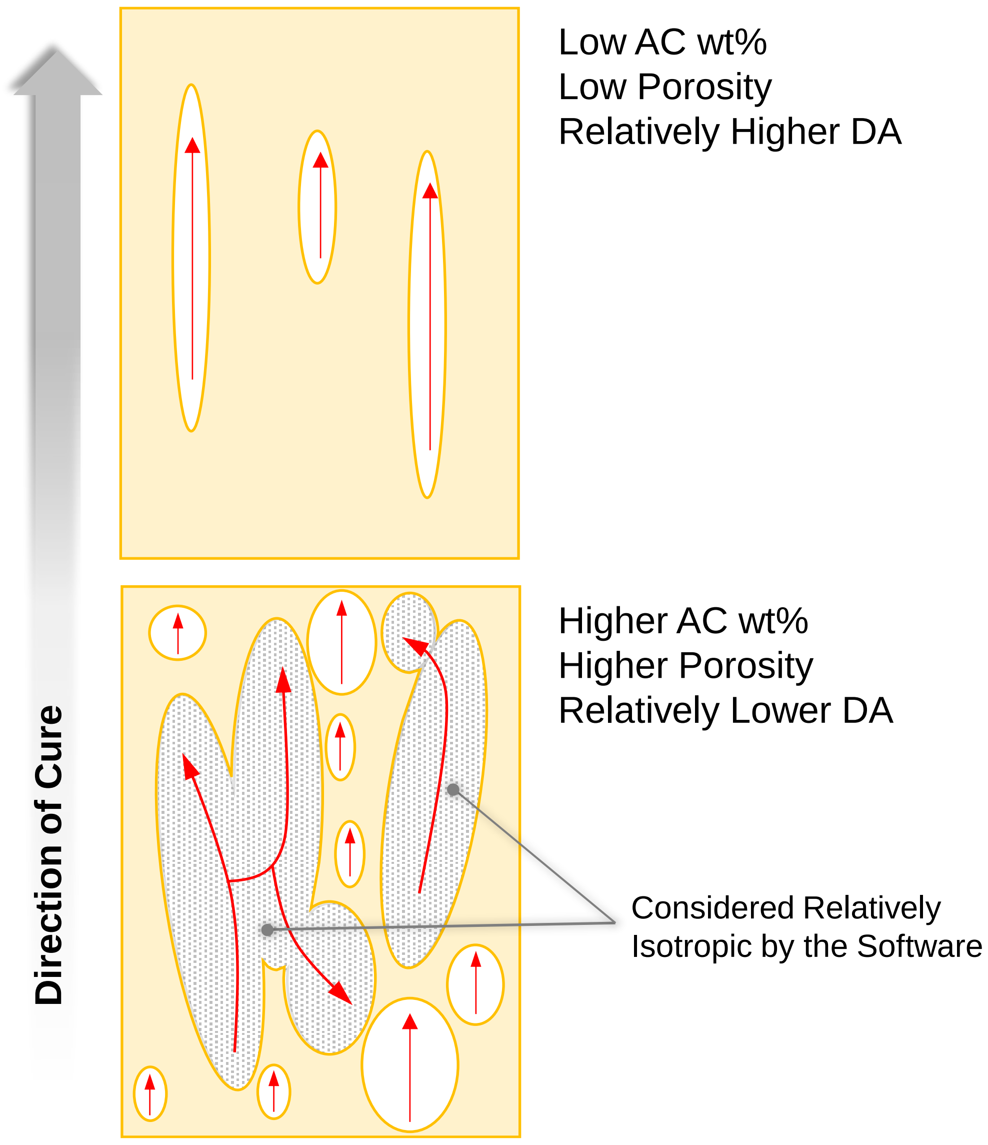

Supplementary Figure 7. Degree of anisotropy (DA) were measured using the Bruker CTAn analyzer. Briefly, DA values are obtained as eigenvalues by the mean intercept length (MIL) calculation. With increasing AC wt%, the formed pore walls were interconnected to each other by which are indicated as one large pore with various directions on the software.

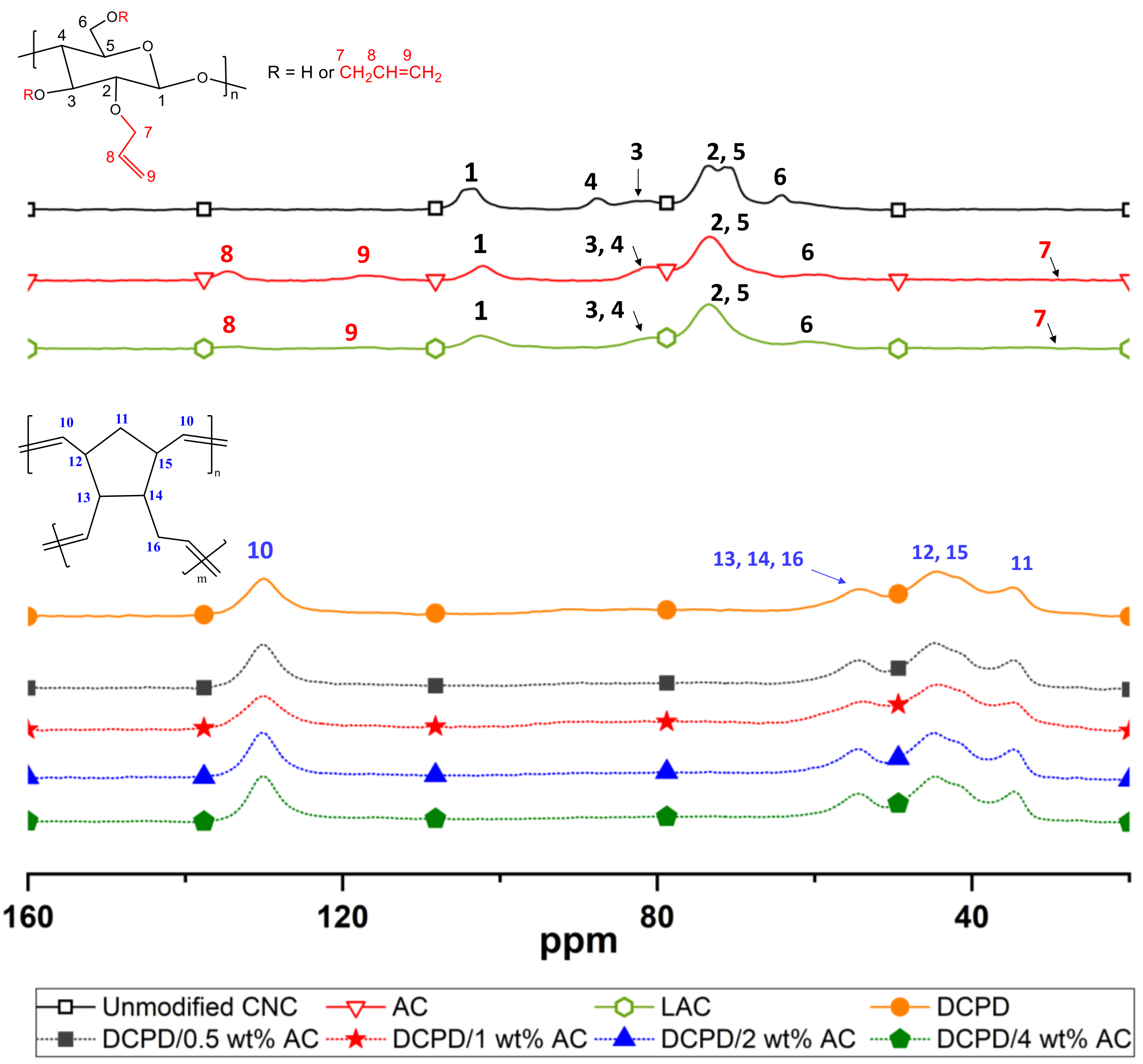

Supplementary Figure 8. ^{13}C CP-MAS solid NMR spectra of unmodified CNC, AC, LAC, neat DCPD, and DCPD/AC foams of varying AC wt% are displayed. Although increasing wt% of AC were introduced to DCPD, no chemical shifts or intensity changes at around 130 ppm (C=C bond) were observed compared to the neat DCPD peak.

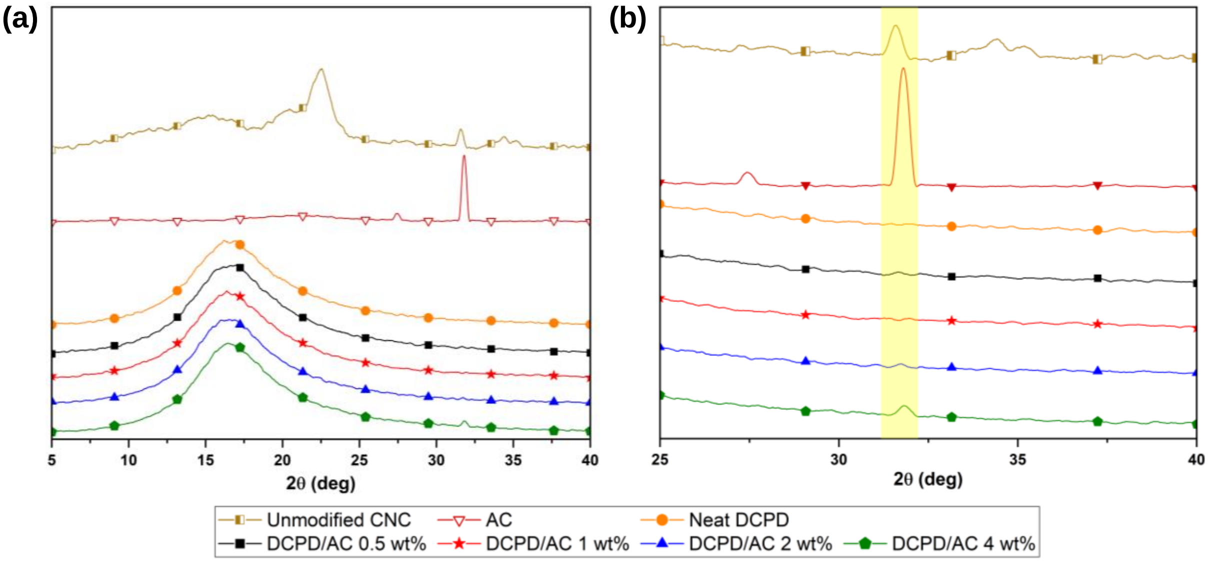

Supplementary Figure 9. (a – b) The X-ray diffraction (XRD) patterns of unmodified CNC, AC, neat DCPD, and DCPD/0.5 – 4wt% AC foams are shown. With increasing AC wt%, peak intensity pertaining to the cellulose (004) plane became increasingly apparent, suggesting the unlikeliness of ACs being interacted with DCPD undergoing ROMP while being present in the same reaction pot.

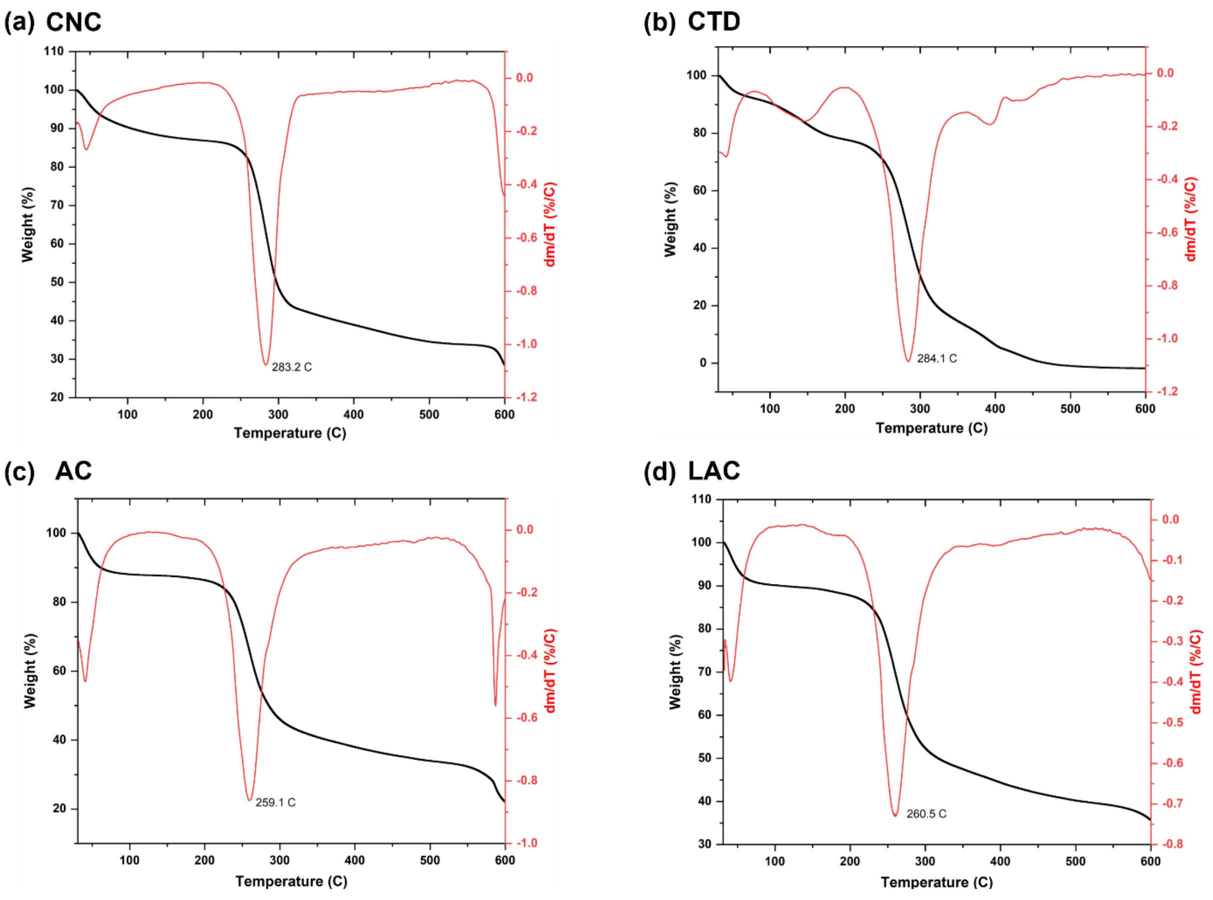

Supplementary Figure 10. Thermogravimetric analysis of **(a)** unmodified CNC, **(b)** CTD, **(c)** AC, and **(d)** LAC. These reactants displayed robust thermal stability at temperatures that were significantly higher than the maximum reaction temperatures observed in this study.

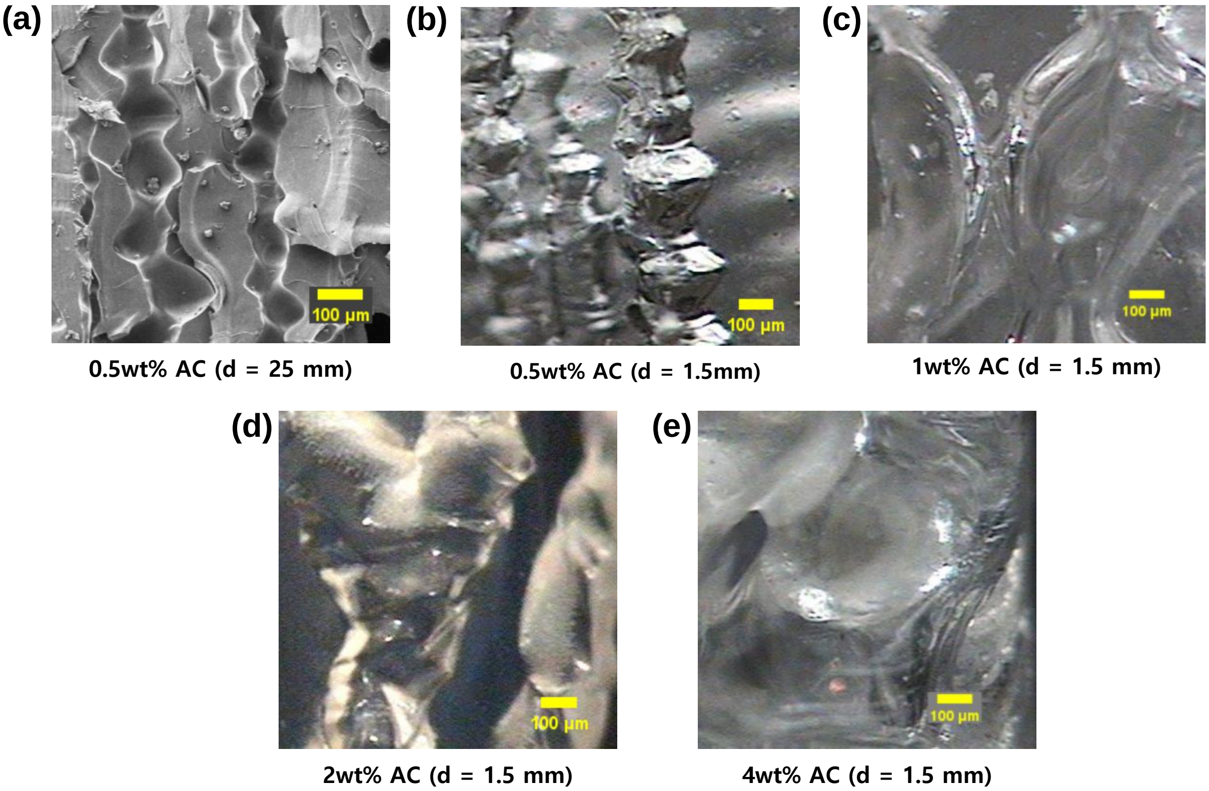

Supplementary Figure 11. (a) An image of a cross section of DCPD/0.5wt% AC monolith produced in a d = 25 mm glass vial obtained from the FE-SEM analysis. **(b)** Digital optical microscope image (x 0.63) of the DCPD/0.5wt% AC mixture cured in a d = 1.5 mm capillary tube. **(c-e)** Digital optical microscope images (x 0.63) of DCPD/1-4wt% AC showed that with increasing AC wt%, the formed millichannels merged with each other thereby forming wider pores.

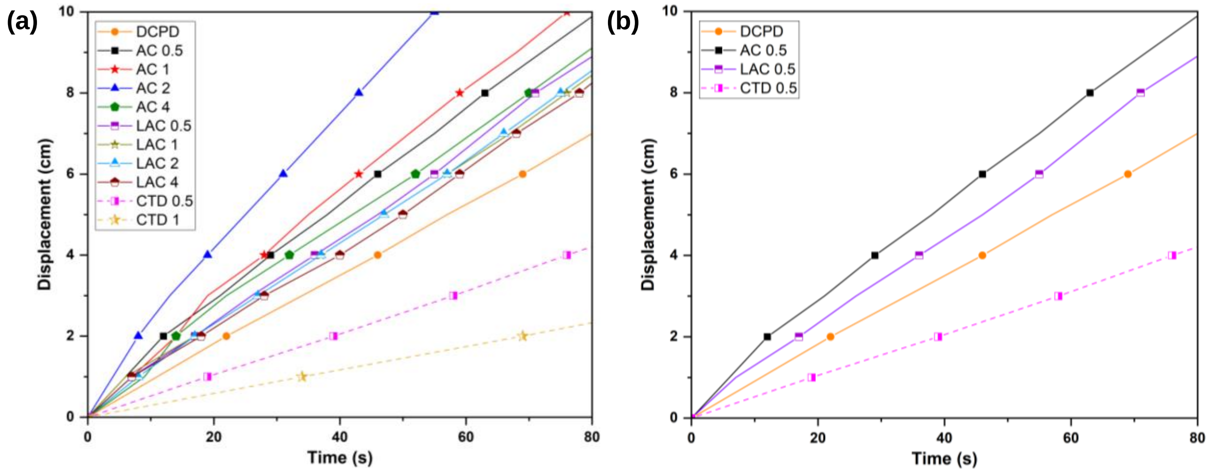

Supplementary Figure 12. Front displacement (*x*) vs. time (t) plots of **(a)** neat DCPD, DCPD/0.5 – 4wt% AC, DCPD/0.5 – 4wt% LAC, and DCPD/0.5, 1wt% CTD reactions were obtained. When comparing the smallest wt% of each cellulose variants, **(b)** a significant difference in the measured reaction kinetic was observed.

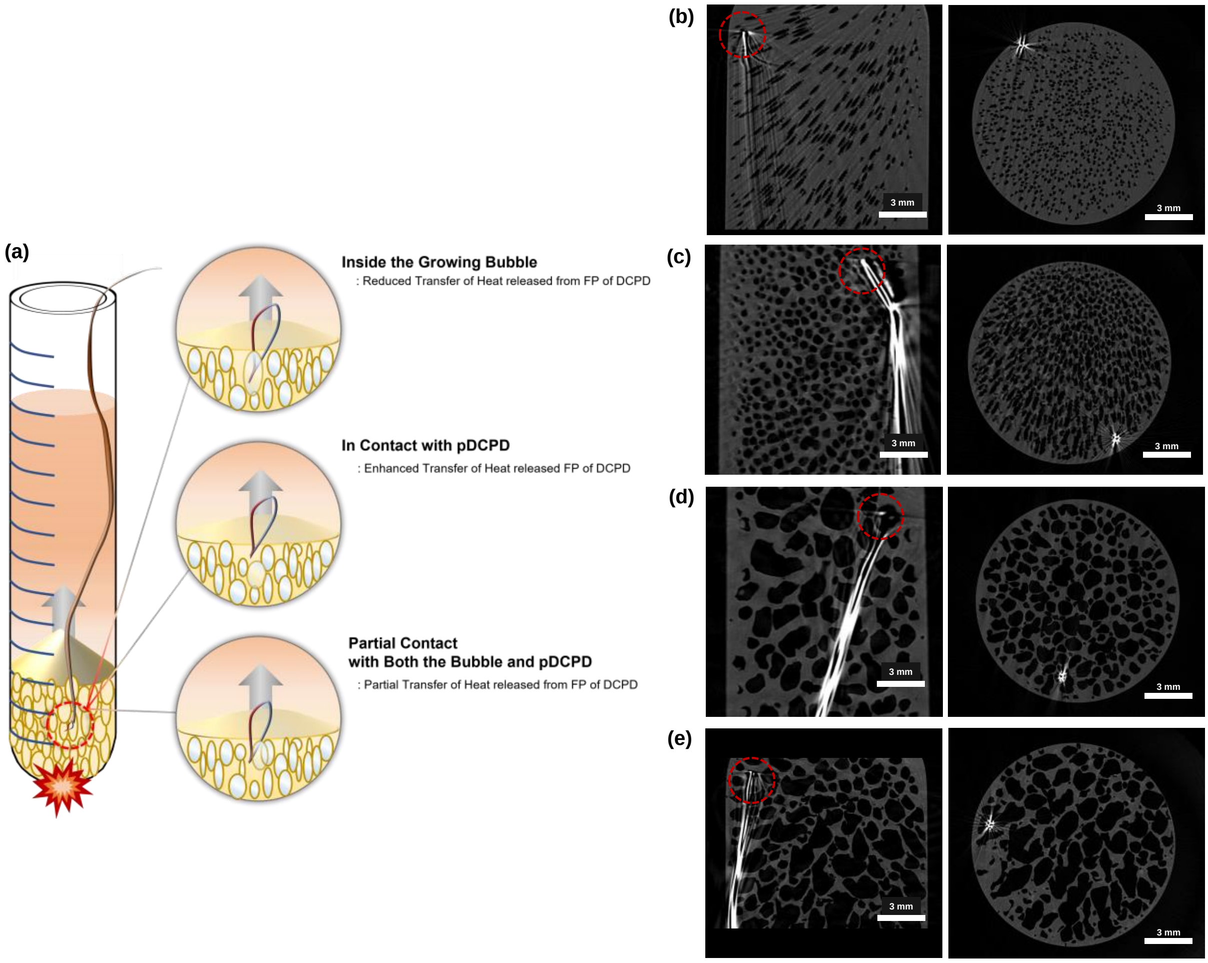

Supplementary Figure 13. (a) A schematic is shown in support of our presumption that the stochastically growing nature of bubbles led to the large standard deviation due to the incomplete contact between the thermocouple hot junction and the polymerizing monomer. **(b-e)** μ-CT images of the polymerized foams showed that the hot junctions of the thermocouples were sometimes located inside the pores (dark areas) or in contact with the polymer (grey area).

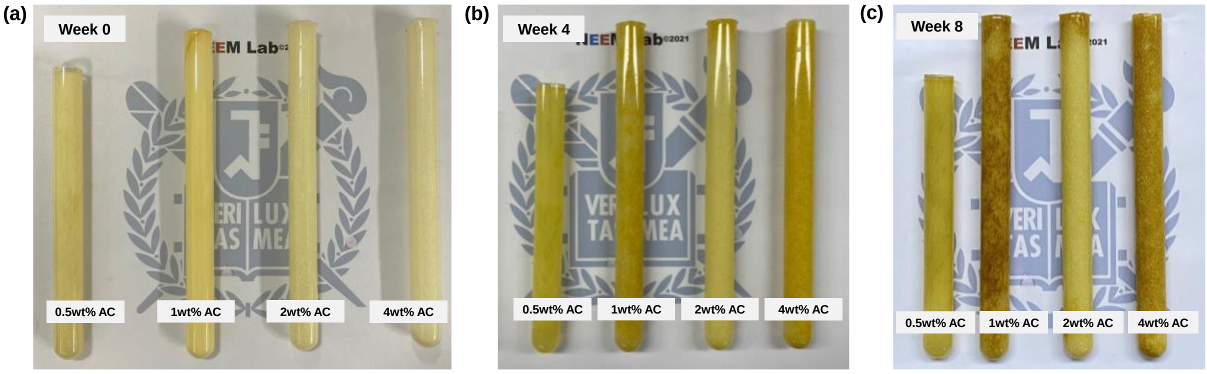

Supplementary Figure 14. Oxidation of the produced DCPD/AC foams in ambience were observed over 8 weeks. The more the foams were oxidized, the browner they became. However, DCPD/2wt% AC exhibited a relative resistance to oxidation.

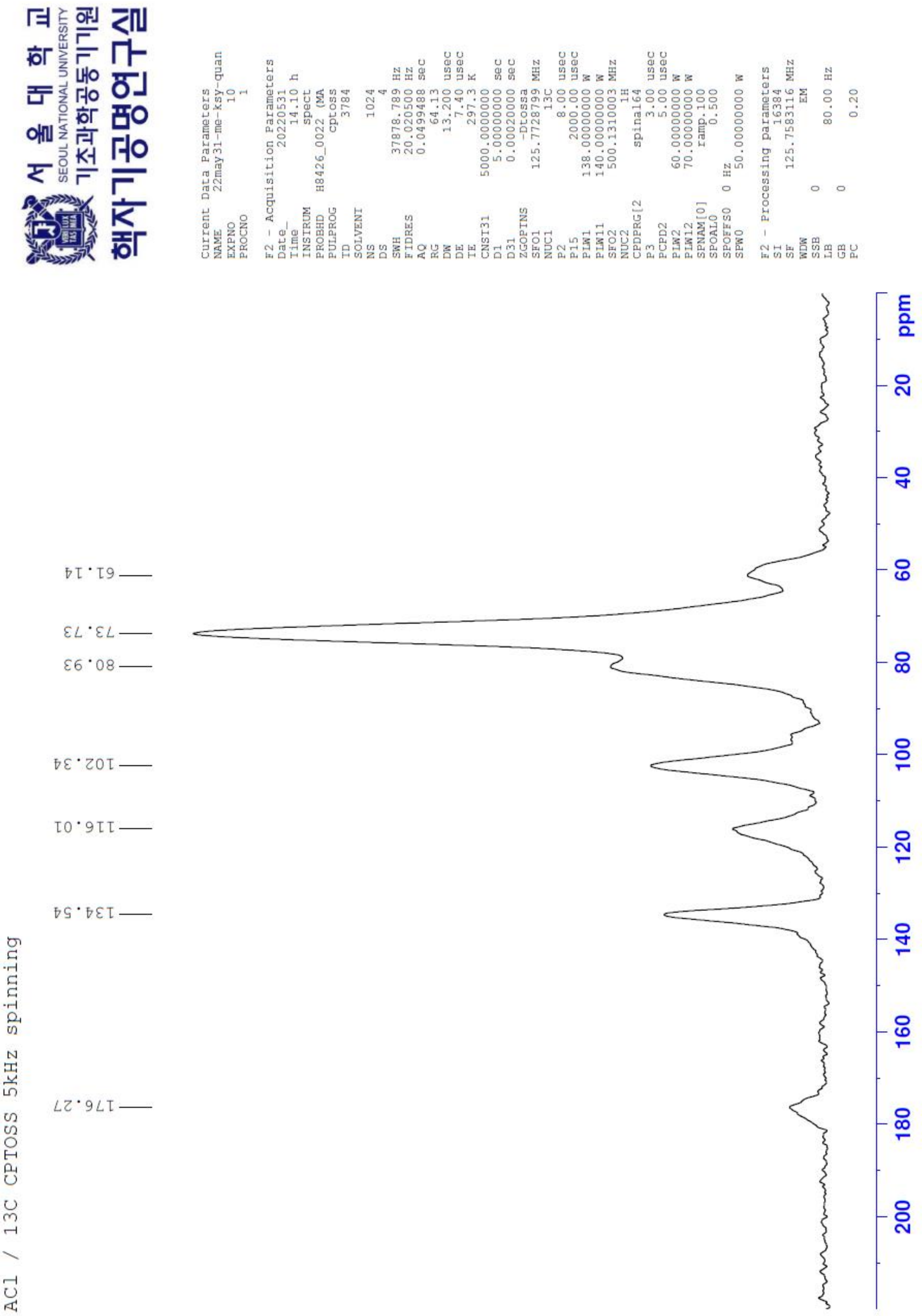

Supplementary Figure 15. An unedited ^{13}C CP-MAS Solid NMR spectrum of AC

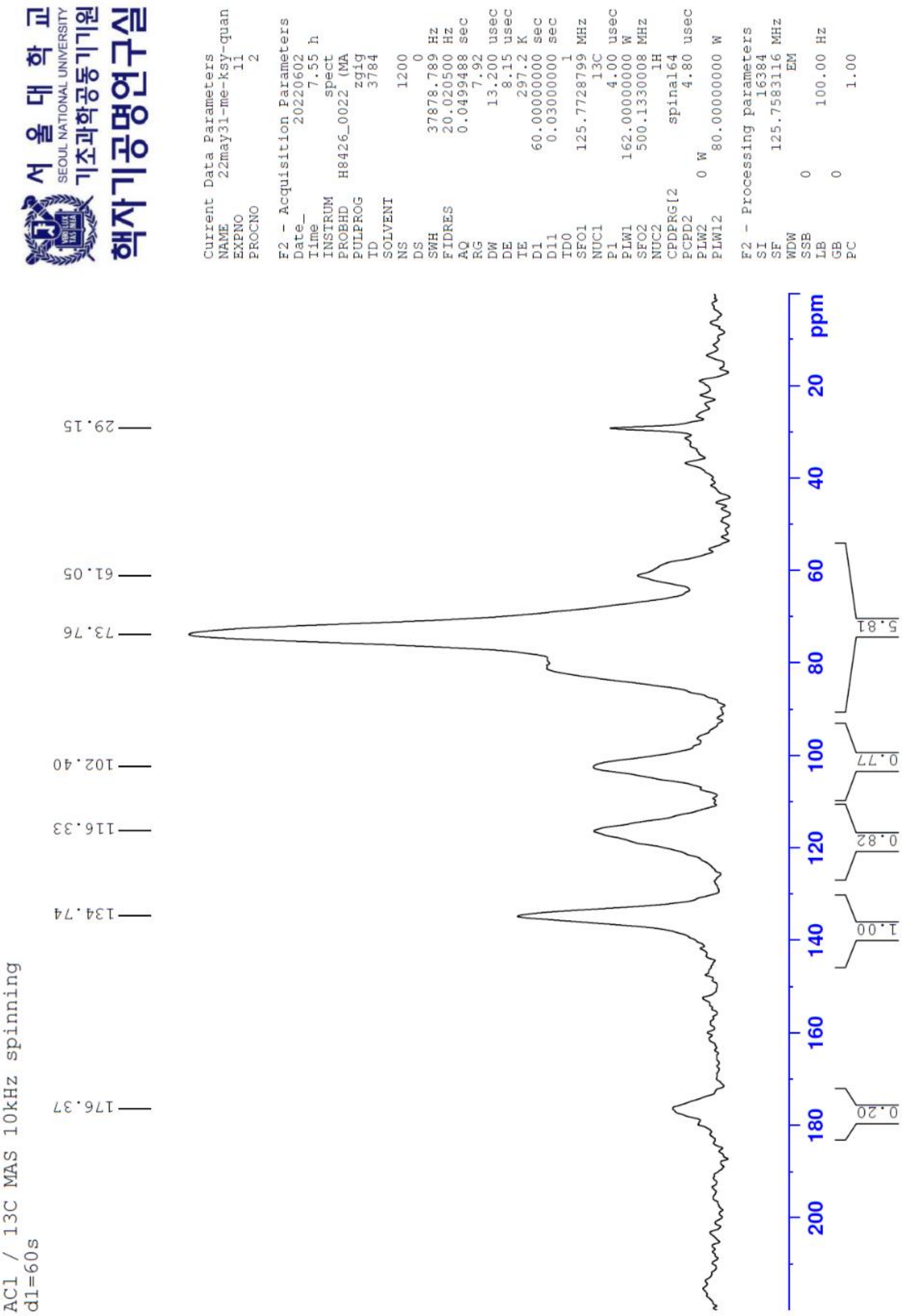

Supplementary Figure 16. An unedited ^{13}C IG-Solid NMR spectrum of AC

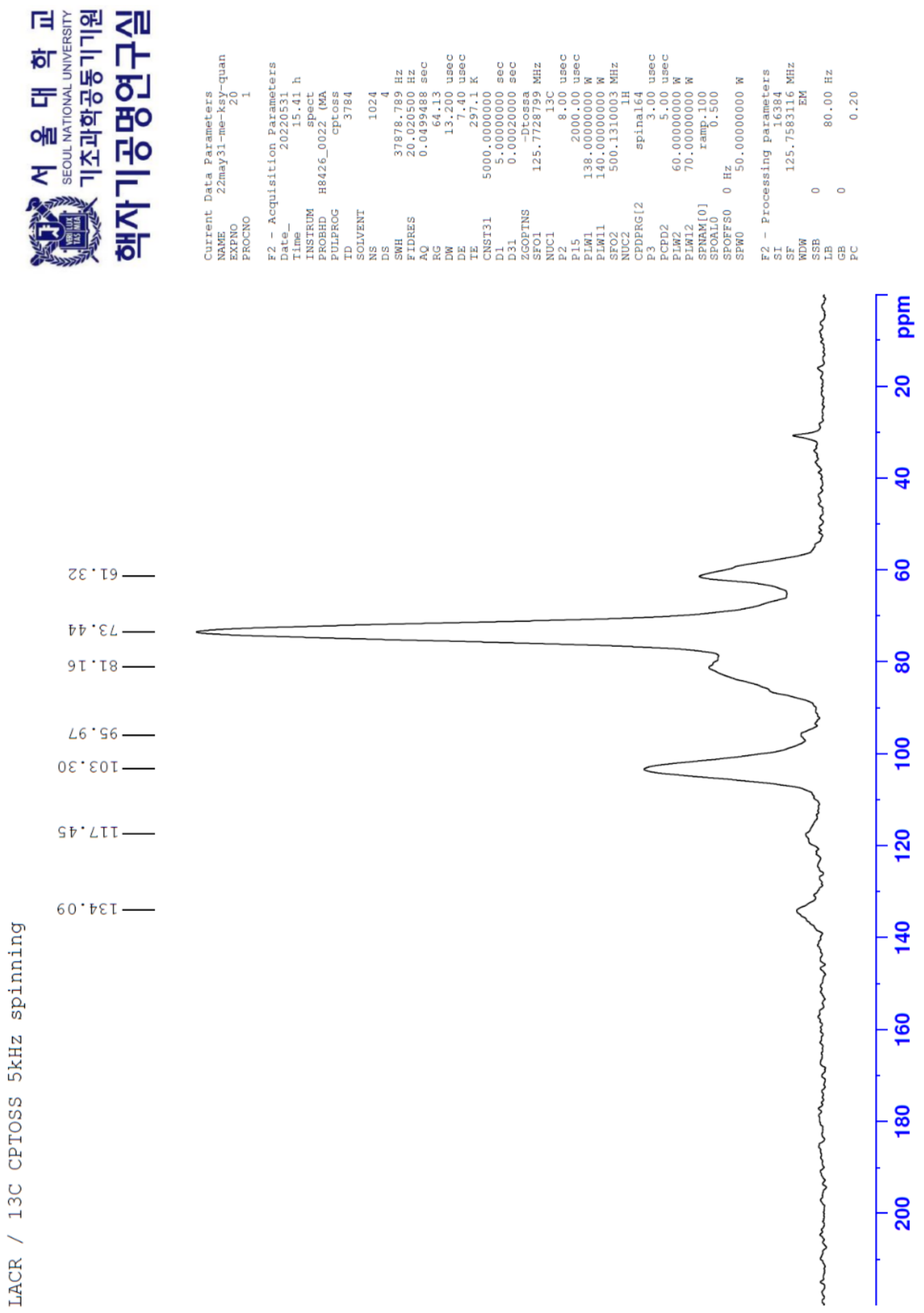

Supplementary Figure 17. An unedited ^{13}C CP-MAS Solid NMR spectrum of LAC

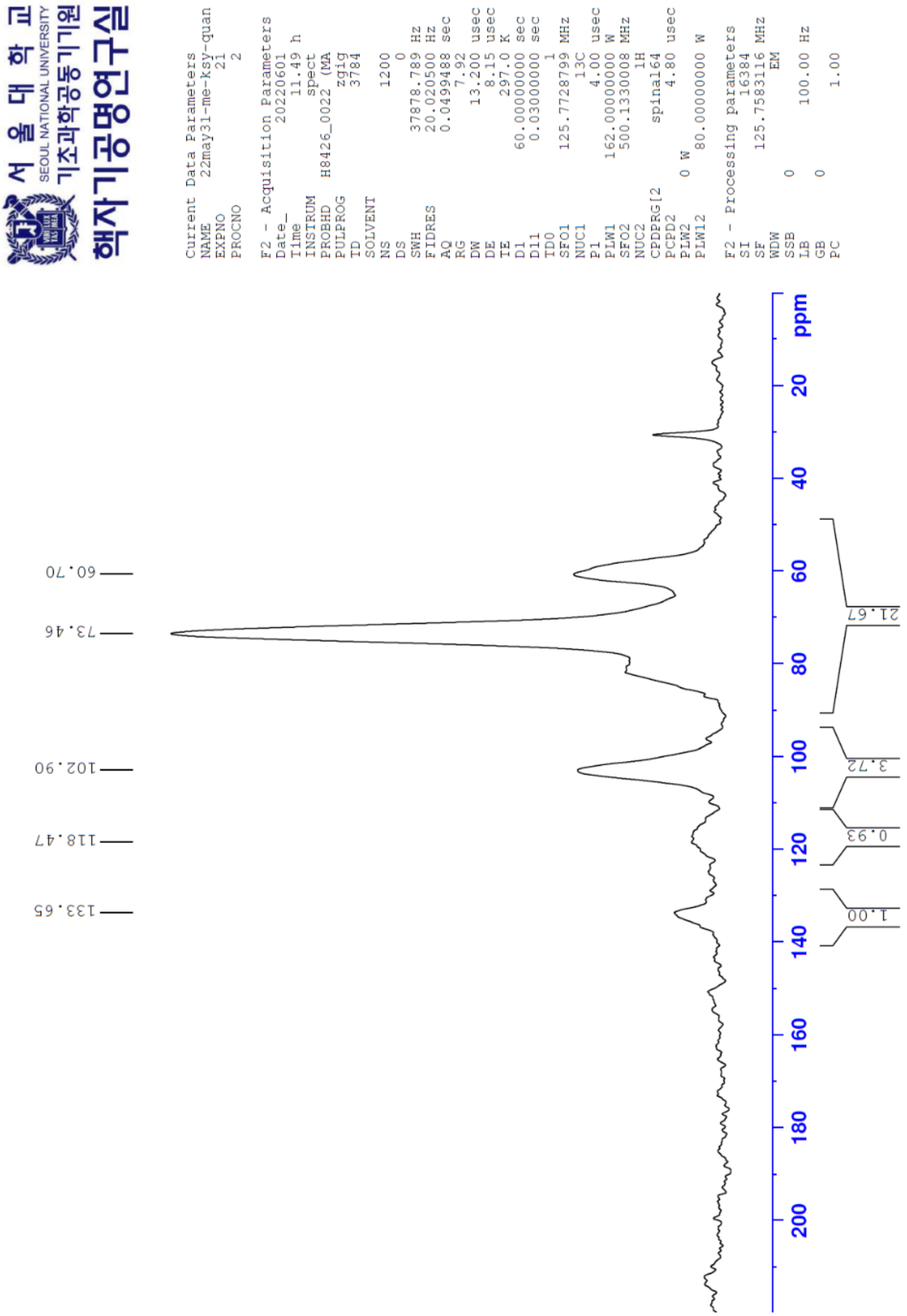

Supplementary Figure 18. An unedited ^{13}C IG-Solid NMR spectrum of LAC
