# Supplementary material for: Frontal polymerization-triggered simultaneous ring-opening metathesis polymerization and cross metathesis affords anisotropic macroporous dicyclopentadiene cellulose nanocrystal foam
Source: Commun Chem. 2022 Oct 7;5:119. doi: 10.1038/s42004-022-00740-1 (PMC9814902; doi:10.1038/s42004-022-00740-1)
Supplement: Supplementary file 3 — Description of Additional Supplementary Files [file 42004_2022_740_MOESM3_ESM.docx]

Description of Additional Supplementary Files

**File name:** Supplementary Video 1

**Description:** Frontal polymerizations of DCPD/0.5 wt% AC

**File name:** Supplementary Video 2

**Description:** Frontal polymerizations of DCPD/1 wt% AC

**File name:** Supplementary Video 3

**Description:** Frontal polymerizations of DCPD/2 wt% AC

**File name:** Supplementary Video 4

**Description:** Frontal polymerizations of DCPD/4 wt% AC
